# Data on cost analysis of drilling mud displacement during drilling operation

**DOI:** 10.1016/j.dib.2018.05.075

**Published:** 2018-05-18

**Authors:** Emeka Emmanuel Okoro, Adewale Dosunmu, Sunny E. Iyuke

**Affiliations:** a Petroleum Engineering Department, Covenant University Ota, Nigeria; b Petroleum Engineering Department, University of Port Harcourt, Nigeria; c Chemical Engineering Department, Petroleum Training Institute, Nigeria

## Abstract

The focus of this research was to present a data article for analyzing the cost of displacing a drilling fluid during the drilling operation. The cost of conventional Spud, KCl and Pseudo Oil base (POBM) muds used in drilling oil and gas wells are compared with that of a Reversible Invert Emulsion Mud. The cost analysis is limited to three sections for optimum and effective Comparison. To optimize drilling operations, it is important that we specify the yardstick by which drilling performance is measured. The most relevant yardstick is the cost per foot drilled. The data have shown that the prices for drilling mud systems are a function of the mud system formulation cost for that particular mud weight and maintenance per day. These costs for different mud systems and depend on the base fluid. The Reversible invert emulsion drilling fluid, eliminates the cost acquired in displacing Pseudo Oil Based mud (POBM) from the well, possible formation damage (permeability impairment) resulting from the use of viscous pill in displacing the POBM from the wellbore, and also eliminates the risk of taking a kick during mud change-over. With this reversible mud system, the costs of special fluids that are rarely applied for the well-completion purpose (cleaning of thick mud filter cake) may be reduced to the barest minimum.

**Specifications Table**

**Table:** 

Subject area	Petroleum Engineering
More specific subject area	Drilling Engineering
Type of data	Table, figure
How data was acquired	An oil well in Niger-Delta region, it was planned to be drilled as an Appraisal and Development oil well to a depth of 9513 ft in four hole sections. These are the 20” (stove pipe), 16”, 12 ¼” and 8 ½” hole sections. The cost of additives and chemicals used were received on March, 2018 from Best Land and Sea (BLS) Service.
Data format	Raw, Analyzed
Experimental factors	The prices are based on build cost for a certain mud weight and daily maintenance expense.
Experimental features	To optimize drilling operations, it is important to specify the yardstick by which drilling performance is measured. For the data set, the most relevant yardstick is the cost per foot drilled; which can be used in drilling contracts.
Data source location	Rivers State, Nigeria.
Data accessibility	Data are available within this article.
Related research article	None.

**Value of the data**

• These data describe the volume and material estimate needed for each hole section and the type of mud necessary to achieve smooth drilling operation in each hole section.
• The data showed the cost, the quantity of materials and sequence at which these materials are applied to achieve optimum displacement.
• These data can be used to study the economic analysis of new mud systems proposed by researchers and also help to compare if these mud systems are economically viable.
• These data can also be used to analyze and predict prices and/or build cost for drilling mud systems for a certain mud weight and daily maintenance expense.
• The data reveals that cost varies according to the different mud types and are dependent on the base fluid phase.

## 1 Data

The type of drilling fluid systems and the volume of drilling fluid needed for each hole section is summarized in Table 1. The well was spudded with Bentonite/Polymer mud system. The mud was then converted to KCl/Polymer mud system by the addition of Pre-hydrated KCl into the system. The 12 ¼” hole section was drilled with Pseudo Oil Based Mud (POBM) system. The 8 ½” development hole section was drilled with Non-Aqueous Fluid system (NAF).

**Table 1:** Summary of mud types used during the drilling operation.

**Components**	**Well sections**			
Open hole diameter	24 ˝	16 ˝	13 5/8 ˝	12 1/4 ˝
Casing/ liner diameter	16 ˝	13 5/8 ˝	12 1/4 ˝	8 1/2 ˝
Description	Surface/conductor	Top hole	Intermediate	Reservoir section
Conventional mud type	SPUD	KCL	POBM	NAF

The volume of drilling fluids needed for three sections was estimated and presented in Table 2, Table 3, Table 4. The cost and how successful an oil or gas well will be completed depends to a substantial extent, on the properties and characteristics of the drilling fluid (Amorin et al. [1]). A considerable number of drilling fluid formulations have been developed by researchers and the selection of the best fluid to meet the formation to be drilled conditions will minimize well costs.

**Table 2:** Volume estimate for the bentonite/ polymer mud system (SPUD mud).

**S/N**	**Section**	**Internal diameter, ID**	**ID square**	**Depth (ft)**	**Conversion factor**	**Volume of mud**
1	Surface volume					**600**
2	24" Casing to 400 ft	24	576	400	1029	223.9067055
3	16" Open hole to 2000ft	16	256	1600	1029	398.0563654
4	Wash out 20%					244.3926142
5	Losses behind casing 20%					0
6	PIT/transit losses 5%					61.09815355
7	Hole enlargement					39.80563654
8	**Total volume (bbl)**					**1567.259475**

**Table 3:** Volume estimate for KCl/ polymer mud.

**S/N**	**Section**	**Internal diameter, ID**	**ID square**	**Depth (ft)**	**Conversion factor**	**Volume of mud**
1	Surface volume					**600**
2	24" Casing to 400 ft	24	576	400	1029	223.9067055
3	16" Open hole to 5010 ft	16	256	4610	1029	1146.899903
4	Wash out 20%					394.1613217
5	Losses behind casing 20%					394.1613217
6	PIT/transit losses 5%					98.54033042
7	Hole enlargement					114.6899903
8	**Total volume (bbl)**					**2972.359572**

**Table 4:** Volume estimate for POBM mud.

**S/N**	**Section**	**Internal diameter, ID**	**ID square**	**Depth (ft)**	**Conversion factor**	**Volume of mud**
1	Surface volume					**600**
2	13 3/8" casing @ 5000 ft	13.375	178.89063	5000	1029	869.2450194
3	12 1/4" open hole to 9386 ft	12.25	150.0625	4386	1029	639.625
4	Wash out 20%					421.7740039
5	Losses behind casing 20%					421.7740039
6	PIT/transit losses 5%					105.443501
7	Hole enlargement					63.9625
8	**Total volume (bbl)**					**3121.824028**

The cost for drilling a typical well may be constant when drilled without any instability case. Instability during a drilling operation in wells can quickly escalate cost dramatically (Okoro et al. [2]). The materials and their cost for each drilling fluid systems are presented in Table 5, Table 6, Table 7.

**Table 5:** Spud mud material estimate.

**Products**	**Unit size (kg)**	**Cost/unit (USD)**	**Conc.: lbs/bbl**	**Units**	**Total cost (USD)**
Bentonite (1mt)	1000	605	25	18	10890.00
Caustic Soda	25	75	0.25	8	600.00
Soda Ash	25	32	0.25	8	256.00
CMC HV	25	124	2	57	7068.00
CaCO3 fine	50	21	10	143	3003.00
CMC LV	25	124	2	57	7068.00
Drilling surfactant	55	945	0.5	7	6615.00
Ultra seal	25	100	2	57	5700.00
Mica	25	36.3	2	57	2069.10
**Total cost (USD)**					**43269.10**
**Total volume (bbl)**					**1567.26**
**MD (ft)**					**2000.00**

**Table 6:** KCl/ polymer mud material estimates.

**Products**	**Unit size (kg)**	**Cost/unit (USD)**	**Conc.: lbs/bbl**	**Units**	**Total cost (USD)**
Bentonite (1mt)	1000	605	18	25	15125.00
Caustic soda	25	75	0.25	14	1050.00
Soda ash	25	32	0.25	14	448.00
PAC-R	25	150	2	108	16200.00
Borhamyl starch	25	62.5	4	216	13500.00
PAC-L	25	150	1	54	8100.00
XCD polymer	25	312	1	54	16848.00
KCl (1mt)	1000	1450	21	29	42050.00
CaCO3 F/M	50	21	10	270	5670.00
Soltex	25	108	4	216	23328.00
Surfactant (gal)	1	945	1	8	7560.27
Mica fine	25	36.3	2	108	3920.40
Ultra seal LCM	25	100	2	108	10800.00
Barite (1mt)	1000	400	50	68	27200.00
Paraffin (bbl)	36	266.65	2	24	6399.60
**Total cost (USD)**					**183074.27**
**Total volume (bbl)**					**2972.36**
**MD (ft)**					**5010.00**

**Table 7:** POBM mud system material and cost estimate.

**Product**	**Unit size**	**Unit price (USD)**	**Conc.: ppb/bbl**	**Sxs/drm/bbl**	**Total cost (USD)**
EDC 99 DW (1 bbl)	0.5	266.65	0.64	8	2133.20
Primary emulsifier (gal)	4	535	6	49	26215.00
Secondary emulsifier (gal)	4	715	3	24	17160.00
Organophilic clay (kg)	25	88	8	454	39952.00
Lime (kg)	25	13.5	4	227	3064.50
Soltex (kg)	25	108	4	227	24516.00
CaCO3 F/M (kg)	50	21	8	227	4767.00
Barite (mt)	1000	400	219	311	124400.00
Calcium chloride (kg)	25	24.35	30	1700	41395.00
Rheology modifier (gal)	4	810	1	8	6480.00
Wetting agent (kg)	55	590	0.85	22	12980.00
Fresh water (1 bbl)	0.5	0	0.236	3	0.00
**Total cost (USD)**					**303062.70**
**Total volume (bbl)**					**3122.08**
**MD (ft)**					**9386.00**

**Fig. 1:**
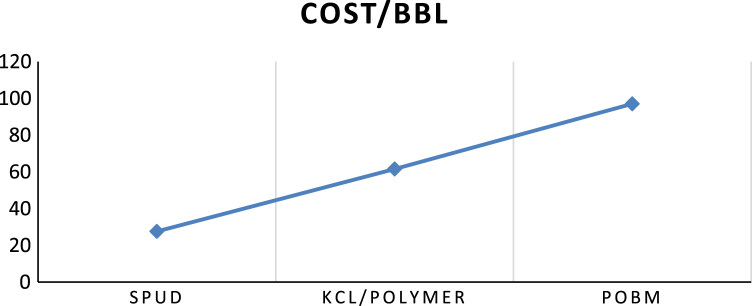
Cost per barrel for each mud systems.

**Fig. 2:**
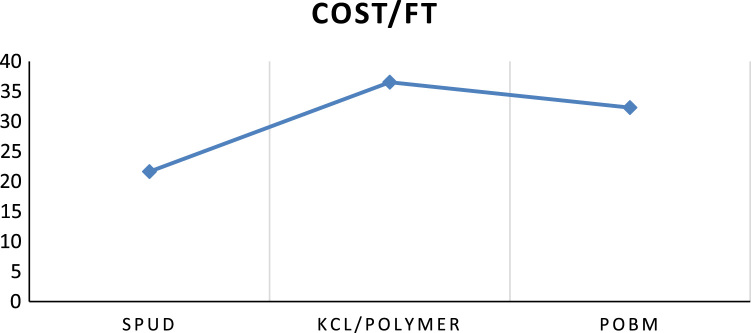
Cost per feet drilled for each mud systems.

Fig. 1, Fig. 2 illustrates the cost per barrel and cost per feet drilled respectively for the mud systems used in the drilling operations.

The build cost for a drilling fluid system is the price for the individual components and mixing requirements. The total build cost includes purchasing the initial drilling systems materials and the expenses involved with conditioning the drilling mud system in the well as it is drilled.

## 2 Experimental design, materials, and methods

The water-based mud in the wellbore from the previous hole section is displaced and replaced with POBM drilling fluid. The first step is to lower the viscosity and gel strength of the water-based mud. The suggested method is to dilute the fluid with water to obtain a low rheology (Patel [3]). The optimal thinning of the water-based mud will dictate how easy the mud will be displaced out of the hole. The spacer is pumped first, followed by the POBM mud at maximum pump rate to get the mud in the annulus moving (Table 8).

**Table 8:** 500 bbls sweep and seal pill formulation.

**Products**	**Unit size (kg)**	**Cost/ unit (USD)**	**Concentration (ppb)**	**Units**	**Total cost (USD)**
CaCO_3_ fine	50	21	20	109	2289.00
CaCO_3_ medium	50	21	10	55	1155.00
Soltex	25	108	4	44	4752.00
OBM LCM (lb)	50	59	4	22	1298.00
**Total cost**					**9494.00**

After drilling and prior to running completion hardware, the fluid in the borehole is often displaced to a water-based completion fluid, usually a solution of various salts. During this displacement, chemical washes and viscous spacers are placed in the solution to make surfaces water- wet, while helping to remove oil mud and residual oil-wet material from the borehole (Ali et al. [4]).

The viscosity and gel strengths of the POBM are low prior to displacement. The suggested method was to dilute the fluid with premix, base fluid or a thinner to obtain the low rheology if this is necessary. The optimal thinning of the POBM fluid will dictate how easy the mud will be displaced out of the hole.

The volume of drilling fluid needed for each section was obtained using the equation below:

(1) $$\mathrm{Volume}\;(\mathrm{bbl})=\frac{{\mathrm{ID}}^{2}}{1029}\times D$$

Where,

ID = Hole Internal Diameter, inch
D = Hole Depth, ft

Eqs. (2), (3) were used to estimate the product units needed in gallons and kilogram respectively;

(2) $$\mathrm{Gallons}=\frac{\mathrm{ppb}\times \mathrm{volume\backslash of\backslash mud}\;(\mathrm{bbl})}{\mathrm{Specific\backslash Gravity}\times 8.33\mathrm{ppg}}$$

(3) $$\mathrm{Unit}\;(\mathrm{kg})=\frac{\mathrm{ppb}\times \mathrm{Volume\backslash of\backslash mud}}{\mathrm{Material\backslash Unit}\;\left(\mathrm{kg}\right)\times 2.205}$$

Eq. (4) was used to convert the quantity of additives used from lb/bbl to sxs:

(4) $$\frac{\frac{\mathrm{xlb}}{\mathrm{bbl}}\times \mathrm{Required\backslash Volume}\;(\mathrm{bbl})}{\mathrm{Unit\backslash Size X}2.205}=\mathrm{Number\backslash of\backslash sxs}$$

Where,

sxs = Sacks
lb/bbl = Pound per barrel
bbl = Barrel
