# Supplementary material for: Data on cost analysis of drilling mud displacement during drilling operation
Source: Data Brief. 2018 May 18;19:535–41. doi: 10.1016/j.dib.2018.05.075 (PMC5997905; doi:10.1016/j.dib.2018.05.075)
Supplement: Supplementary file 1 — Supplementary material [file mmc1.doc]

May 10, 2018

Manuscript No. DIB-D-18-01079

Dear Managing Editor,

There is no concern raised about the reviewer’s comment. All issues requested and identified have been effected and highlighted in the reversed manuscript.

The authors appreciate the contributions and state that there is no conflict of interest.

I look forward to hearing from you soon.

Yours sincerely,

Dr. Okoro Emeka Emmanuel

FOR: The Authors
